# Introductory Lecture to a Course of Lectures on the Practice of Physic, to Be Delivered at the Medical School in Aldersgate Street

**Published:** 1836-01

**Authors:** 


ART. IX. — *Introductory Lecture to a Course of Lectures on the Practice of Physic; to be delivered at the Medical School in Aldersgate street.* By MARSHALL HALL, M.D. F.R.S. L. and E., &c. —*London, undated.* 8vo. pp. 55.

*Syllabus of Lectures on the Diseases of the Nervous System; to be delivered in the Aldersgate School of Medicine.* By MARSHALL HALL, M.D. &c. &c.—*London,* 1835. 8vo. pp. 19.

*An Introductory Address, delivered at the commencement of the Tenth Session of the Manchester School of Medicine and Surgery, Pine-street.* By JAMES L. BARDSLEY, M.D. &c.—*London,* 1835. 8vo. pp. 47.

*An Address delivered at the First Anniversary Meeting of the Birmingham School of Medicine and Surgery.* By JAMES THOMAS LAW, Chancellor of the Diocess of Litchfield.—*Birmingham,* 1835. 8vo. pp. 21.

*An Introduction to the Study of Practical Medicine; being an Outline of the leading Facts and Principles of the Science, as taught in a Course of Lectures, delivered in the Marischal College of Aberdeen.* By JOHN MACROBIN, M.D., Junior Professor of Medicine in the Marischal College and University of Aberdeen, &c. &c. Part I.—*London,* 1835. 8vo. pp. 226.

*St. Thomas's Hospital Reports.* By JOHN F. SOUTH, Assistant Surgeon. No. I. November, 1835.—*London.* 8vo. pp. 118.

WE are not among those who look with indifference on Introductory Lectures, Syllabuses, Addresses at Medical-School anniversaries, Introductions to the Study of Medicine, and Hospital Reports. In each publication of this kind we see a manifesto of the principles professed, and the views entertained in the School in which it originates, not less characteristic of such School than the clinical lectures contained in so many of our periodical publications are of those who deliver them. If we read with surprise the language in which some of these clinical discourses are delivered,—a language not only unsuited to the teaching of any science, but sometimes such as would scarcely be tolerated in good society; we are able to contrast these with other clinical discourses, at once so eloquent and so descriptive that we seem to hear the lecturer, and to see the very case which suggests his remarks. To the shame of the School of London must it be added, that for examples of the former kind,—truly among many brighter,—we must look almost exclusively to it. The Schools of Edinburgh, Dublin, and Paris, take a higher tone; and the addresses and lectures delivered in the Provincial Schools of England are uniformly earnest, serious, and dignified; worthy, in short, of the importance of the knowledge which in such institutions it is professed to teach. In the works before us, also, we are happy to say, there are no indications of that bad taste which addresses itself to the most ignorant of the crowd of young men who fill the class-rooms of medical teachers. Of various merits and character, each of these works is composed in a style which becomes the teachers, and is suited to the learners, of a liberal science.

The extensive medical learning, the minute observation, the experience of Dr. Marshall Hall, are, of course, conspicuous in his lecture; and those acquainted with his previous publications will recognize the same zeal to promote an accurate diagnosis,—the same valuable remarks enforced concerning the good and bad effects of bloodletting,—and numerous indications of that attentive notice of all the phenomena of sickness which characterizes this eminent physician. If it be objected, that a large portion of the lecture is already well known to most medical readers, it is to be remembered that it is almost all valuable, and that in this discourse it is addressed to students, to whom it is probably new. The remarks on diagnosis are presented in a very striking manner; as, for instance, in the following passages:

"You see this drawing of the exterior part of the abdomen: here are the marks of a seton; here are the marks of cuppings, and here those of leeches: they are absolutely unaccountable! What was the disease in this case, think you? Why, there was no disease at all! Yet it was proposed by one surgeon, and it was pretended to be the wish of the patient, to extirpate the ovarium! In a similar case at Edinburgh, the abdomen was actually laid open, but no ovarial disease existed!! Now, what is the meaning of all this? In the case represented here it was a *fictitious* disease. The patient was a malingerer in the hospitals, and deceived many hospital physicians. In the other case, the surgeon deceived himself." (P. 14.)

Speaking of bloodletting as a diagnostic, he says,

"A gentleman, residing twelve miles from London, was taken with an affection of the head. I was sent for, and found, on enquiry, that he had been bled early, and that he had speedily fainted under the influence of the loss of blood. The case was long one of great anxiety. Another physician was consulted. It was suspected to be '*ramollissement*.' Confiding in the diagnosis afforded by the *early* syncope in the *early* stage of the disease from bloodletting, I continued to hold out hope of recovery. The patient did recover! Now, the hope I felt, and held out, in this case, flowed almost entirely from the confidence I reposed in the diagnostic afforded by the event of bloodletting!

"A few days ago I was consulted by a medical friend in the case of his wife; he feared peritonitis: there had been previous attacks of the same kind; there were great pain and tenderness of the abdomen; the tongue was loaded: I prescribed bloodletting in the upright position; the patient, though young and stout, fainted on losing eight ounces of blood! From this moment there was no obscurity in the diagnosis, no hesitation in reference to the treatment, no interruption in the recovery." (P. 47.)

In these observations we are reminded of the principles laid down in Dr. Hall's Researches relative to the Effects of Loss of Blood, and some of the facts contained in that work are in the lecture expressed by a table showing under what circumstances the *tolerance* of bloodletting is increased or diminished. The degree of tolerance in health being represented by the quantity of fifteen ounces, as the mean quantity of blood which flows before incipient syncope; that of augmented tolerance is represented, in congestion of the brain, by ℥xl-l; in inflammation of serous membranes, by ℥xxx-xl.; of the parenchyma, by ℥xxx.; and of the skin and mucous membranes, by ℥xvj. The diminished tolerance is marked, in fevers and eruptive fevers, by ℥xii-xiv.; and gradually lower in delirium tremens and puerperal delirium, in laceration or concussion of the brain, in intestinal irritation, in dyspepsia and chlorosis, down to cholera, which is marked by ℥vj. In support of these views, Dr. Hall quotes four entire pages from Mr. Wardrop's Lectures.

The greatest portion of this Introductory Lecture is devoted rather to enforcing the views previously published in Dr. Hall's different works, than to the practice of physic in general. The concluding pages exhibit his arrangement of diseases, which does not appear less arbitrary than that of other nosologists. One class of the Diseases *of Systems* (the first of his two great divisions,) consists of dyspepsia, chlorosis, and *hysteria.* Scorbutus is ranged under the hemorrhages. The arrangement of the Diseases *of Organs* (the second great division,) is, however, unexceptionable.

It is, we think, a matter of regret that Dr. Hall's Lecture should be disfigured by some peculiarities, of a nature to prejudice those unacquainted with him against any of his lessons. The chief of these is an apparent disregard, notwithstanding very formal protestations to the contrary, of being intelligible to his hearers. That the dedication of the lecture (to M. Louis,) should be in *French,* may be regarded merely as an injudicious whim; but, that three or four pages of the lecture should be in the French language, consisting of important quotations, is really a fault. For three-fourths of the hearers we venture to say that these instructive passages were as little edifying as if they had been read in High Dutch. Then, in the list of contents, we read "The Méthode Numérique of M. Louis," which means nothing more than what is now known to every English reader as *the numerical method* of that distinguished physician. Even Laennec is read in French to the students, although the translation is in everybody's hands. Why should "entérite" be used for the well-known word enteritis? and what idea will a young man from a country surgery take away from Dr. Hall's lecture, for being told that "a little *cliquetis* in the breathing" may enable him to give a *confident* judgment. The English student will not find this word in Mr. Hoblyn's Dictionary, a work dedicated to Dr. Hall. If the French Dictionary is referred to, it will be discovered that it means nothing more than crepitation. In the passage, however, in which the word *cliquetis* occurs, the reader is *particularly* referred to Researches, p. 50; in which page the sound is called *crepitus;* and, after all, it would seem that râle, rattle, or rhonchus, was what was really heard. Inaccuracies of this kind are very perplexing to a student, and ought particularly to be avoided by a lecturer. It appears, also, to be from an unjust partiality for the French idiom, that Dr. Hall calls anatomy, pathology, and morbid anatomy, *the* anatomy, *the* pathology, and *the* morbid anatomy, which is certainly not English. We mention these blemishes freely; for we know they produce an effect upon many readers detrimental to the author, who, making allowances for some eccentricities of this kind, and perhaps for a restriction of observation to too small a range of subjects for a medical teacher, is highly entitled to the respect of the profession.

Dr. Bardsley's Introductory Address is more general in its subjects than that of which we have just spoken; but its principal object is to explain, at the commencement of the session, the means possessed in the Manchester School of affording an efficient education to medical students. The advantages afforded by the School, which are considerable, are set forth without parade, and without exaggeration; and conveyed in language correct, and even elegant, accompanied by sentiments of a nature to rouse the just ambition, and elevate the views of the students. To Manchester belongs the distinction of having, in 1824, established the first complete provincial medical school in England. With a population of 200,000 persons; an hospital into which nearly 20,000 patients are admitted in a year, including upwards of 4000 accidents; and with such teachers as Turner, Ransome, Dalton, Henry, and Bardsley, its efficiency can only be doubted, we presume, by the College of Surgeons, who have twice, Dr. Bardsley informs us, refused *to acknowledge* the Manchester hospital, or to give it equal privileges with hospitals in London containing not more than fifty beds. A regular system of clinical instruction is established in the Manchester hospital. There is also a Fever hospital in which one hundred patients can be received; although at Manchester, as elsewhere, students have not yet become sensible of the immense importance of making themselves acquainted with the forms and varieties of fever. The medical school possesses museums, models, drawings, engravings, a library, medical societies, and every auxiliary that students can require to stimulate and to assist their industry. Considering all these circumstances, no reasonable person can doubt that the town which has already inscribed in medical annals the names of Percival, White, Ferriar, Bardsley, sen., Simmons, Home, Henry, and Roget, will continue to furnish useful and honourable labourers in all the departments of medical science, by whom the boundaries of each will in all likelihood be increased.

The Rev. Chancellor Law must permit us to notice him among doctors and teachers, in consequence of the apparent kindness and zeal with which he has lent his services to the interests of the Medical School of Birmingham, at the first anniversary meeting of which, held in August, his Address was delivered. We cordially agree with the reverend gentleman in hoping that the profession of which he is an ornament, and the profession of medicine, may "always be found advancing hand in hand in the great Christian duty of contributing, each according to its respective means, to the welfare of the human race." It gratifies us to read the terms in which he speaks of the general character of the members of our profession, which abounds, he is pleased to say, "with gentlemen of the most christian-like tone and temper, and of singular humanity; remarkable alike for the strength,—the correctness,—and the richness of their highly cultivated and Christian minds." We trust the Birmingham students will ever remember this praise, and also, that *they* belong to the profession described in such honourable terms. As we happen to know that this Address was published at the urgent request of the hearers, and with the reluctant consent of Mr. Law, we do not deem it a proper object of criticism; but we may sincerely express our admiration of the judgment with which its topics are selected, and the delicacy with which they are treated. The preliminary education of students, the reputation of the teachers in the Birmingham school,—the zeal and perseverance of its founder, Mr. Sands Cox,—the munificence of the patrons,—are all touched upon with much discrimination; and there is throughout an indication of warm and generous feelings which every reader must admire. Appended to the Address is the Report of the School, by which it appears that the number of students is ninety; and that not a single student educated at the Birmingham school, during a period of eight years, has been rejected by the Royal College of Surgeons. In the departments of Anatomy, Natural History, Botany, and Mineralogy, the great exertions which have been made are shewn in the extent and beautiful arrangement of the museums. That of anatomy is receiving continual additions. The present Sir Charles Throckmorton, of Coughton Hall, (Warwickshire,) in early life studied medicine; becoming afterward by accident a prisoner in France, during the war, he was permitted (in consequence, we believe, of a connexion between the ancient English family of Throckmorton and that of General Clarke, Duc de Feltre,) to travel to the Pyrenees and Alps; during which journey he made a collection of dried plants, which he has liberally presented to the Botanical museum of the Birmingham School.

A medical library is attached to the Birmingham School, and already contains more than nine hundred volumes; among which are many rare and valuable editions of the ancient medical authors. In this, as in other departments, we believe the School has been materially assisted by the Drs. Johnstone, of Birmingham, so well known for their learning and extensive professional reputation. Altogether the Report is of a most satisfactory description, and the success of the Birmingham School may be considered as fully established.

If the merits of a work were alone to determine the length of our notice of it, that of Dr. MACROBIN would demand a considerable space in these pages. It is itself a most condensed analysis of the present state of our knowledge of congestions, inflammations, hemorrhages, dropsies, and fevers; and therefore scarcely admits of abridgment. It constitutes the first part of a work which, although modestly entitled an Introduction, will rather, we predict, take rank as a Conspectus or Manual of Practical Medicine; not less suited to the practitioner, as a concentrated recapitulation of such practical knowledge as may be considered established, than to the student, as a convenient text-book. In the Fifth chapter, which we have very carefully perused, and which is devoted to the important subject of Fever, we meet in every page with evidences both of the exact knowledge of the author and of his well-exercised judgment: the facts presented to the reader, derived from authentic sources, cannot be read without putting the mind on its guard against hasty generalizations, by shewing the various aspects of fever, and its various consequences, in different circumstances. The practical rules are delivered with great simplicity, and with reference throughout to the pathology of the disease, founded on the best authorities of all countries. We consider it no small praise to say that Dr. Macrobin's manner of treating the various subjects comprehended in this volume continually reminded us of that of one of the most distinguished living professors of Edinburgh, familiar to us in his lectures and his writings,—we mean Dr. Alison; and we were consequently pleased to observe that the author had been one of the assistants in the clinical wards of the Edinburgh Infirmary, and in the hospital at Queensberry House, in 1826-27, when the fever prevailed which is so admirably described by Dr. Alison, in the Edinburgh Medical and Surgical Journal. A better school, or better preceptors, could nowhere be found; and it will indeed be fortunate for Aberdeen, and powerfully redeem the errors of by-gone days, if its school becomes characterized by the same merits. The style of Dr. Macrobin's book is marked throughout by the correctness, the calmness, and the judicious decision which bespeaks the accomplished physician: there are in it no appeals to the prejudices and passions of his hearers; but every page is calculated to awaken the attention, to assist the memory, and to exercise the understanding. These are the strongest and best recommendations such a work can possess for the student of medicine.

Having commenced this notice by some observations on the abuses which in a few instances deform the clinical instructions of London, we have particular pleasure in concluding it by a brief allusion to the First Number of the St. Thomas's Hospital Reports, edited by Mr. South. These are really clinical lessons; and, although, like other lessons of that kind, their chief utility is felt by those who hear rather than those who read them; by those who see the cases, rather than by those who have to imagine them; yet the reader may derive instruction from reports well detailed, and treatment candidly explained. The subjects of the cases in this number are: Syphilitic Eruptions, treated with Hydriodate of Potash; Wounds of Arteries; Extraction of a broken Catheter from the Bladder by the Urethra; Mortification of the Hand and Fore-arm, following Contusion; Dropsy terminating in profuse Diuresis; Retention of Urine, arising from Gonorrhœa; simple Fracture of the Tibia and Fibula, followed by Paralysis of Motion on the right side of the head and face, and of the arm, from deprivation of stimulus; Hydrocele, treated with Seton; Delirium Tremens; Absorption of a Portion of the Cranium, following a contused Wound, with remarkable symptoms of cerebral Disorder. To most of the cases clinical remarks are added, and they have the merit of being strictly clinical; those on delirium tremens perhaps excepted, which branch out into a kind of general lecture; an error in this case pardonable, on account of the interesting observations of Dr. Roots, but certainly in general objectionable. Although not now delivered at the bedside, clinical lectures ought to be carefully based on a case or cases. Nothing can be more out of place in a *clinical* lecture than the disquisitions sometimes printed under that title, and which are, in fact, lectures on the theory and practice of medicine, almost necessarily superficial, and certainly superfluous. To the St. Thomas's Reports these observations do not apply; and we trust the future numbers will contain a somewhat larger proportion of *medical* cases, the study of which, in the hospitals of the English metropolis, is even yet, we suspect, not so popular as the ordinary duties in which the students are to be engaged render it desirable that it should be. Deeply interested as we have been in the contemplation of the rise and progress of the new Schools of London, we retain an ancient feeling of regard for the Borough hospitals. We cannot forget the great names which have rendered them celebrated all over the kingdom. They needed, no doubt, the stimulus of such an age as that in which we live, but they have not been insensible to it; and, with their prodigious resources, nothing but a want of industry or of talent, neither of which are now wanting, can depress them below the rank of their younger and not unworthy rivals.

The Introductory Lecture of Mr. Travers, delivered in October last, and prefixed to the Reports, is full of judicious observations. He exhorts the students to co-operate with their instructors to restore St. Thomas's School to its pristine grandeur and importance; warns them, that, without industry, temperance, and devotedness to the profession, they cannot attain success; reminds them, that the ultimate object of their study is not a licence or diploma, but to render themselves valuable to society; and that they can only do this by constant attention to lectures and hospital-practice. Those so attentive will, he tells them, be better fitted to pass their examinations by a month's revision of their labours than the idle can become by any kind of preparation. He places before them, in an elevated and just light, the importance of the duties that await them, and tells them that, in these days of artifice and delusion, there is no course left to high-minded men, in the unavoidable competition with pretenders, "but to render their superiority of knowledge a mark, a beacon of distinction, by which they shall be known." It is thus, we think, that students ought to be addressed. The period of study is, indeed, as Mr. Travers well expresses it, not a thraldom, but a privilege; and the value of the privilege can only be felt by those whose views are well directed by their teachers; which can never be more efficiently done than when, as in the case of Mr. Travers, the path to honourable success is pointed out by a teacher equally distinguished by his professional eminence and his estimable personal character.

